# The effect of high‐fat versus high‐carb diet on body composition in strength‐trained males

**DOI:** 10.1002/fsn3.2204

**Published:** 2021-03-11

**Authors:** Michał Wrzosek, Jakub Woźniak, Dariusz Włodarek

**Affiliations:** ^1^ Department of Dietetics Institute of Human Nutrition Sciences Warsaw University of Life Sciences (WULS—SGGW) Warsaw Poland

**Keywords:** body composition, body weight, high‐carbohydrate diet, high‐fat diet, strength sports

## Abstract

Low‐fat, high‐carb (LFHC) and low‐carb, high‐fat (LCHF) diets change body composition as a consequence of the reduction of body fat of overweight persons. The aim of this study is the assessment of the impact of LFHC and LCHF diets on body composition of men of a healthy body mass who do strength sports while maintaining the appropriate calorific value in a diet and protein intake. The research involved 55 men aged 19–35, with an average BMI of 24.01 ± 1.17 (min. 20.1, max. 26.1). The participants were divided into two groups following two interventional diets: high‐fat diet or high‐carb diet, for 12 weeks. The body composition of the participants was measured using bioimpedance. After the 12‐week‐long experiment based on the low‐carbohydrate diet, a significant body mass reduction of 1.5% was observed. In the group, following the LFHC diet, the parameters did not significantly change. In the group following LCHF diet, the body fat reduction of 8.6% from 14 (6.7–19.8) kg to 12.7 (3.9–19.2) was reported (*p* = 0.01) (in the absolute value of 1.2 kg). However, also in the LFHC group, the body fat mass was significantly reduced, that is, by 1.5% (*p* = 0.01) (by 0.4 kg). Nevertheless, it is worth emphasizing that despite significant changes within the groups, these changes were not statistically significant between the groups. Diets with different carbohydrate and fat intake and the energy value covering the energy needs of men training strength sports have similar impact on changes in body composition.

## Graphical abstract

Low‐fat, high‐carb (LFHC) and low‐carb, high‐fat (LCHF) diets change body composition as a consequence of the reduction of body fat of overweight persons. Despite significant changes within the groups, these changes were not statistically significant between the groups. Diets with different carbohydrate and fat intake and the energy value covering the energy needs of men training strength sports have similar impact on changes in body composition.

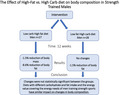

## 1 INTRODUCTION

An optimally balanced diet is one of the most critical factors that positively influence athletic performance. Experts show how athletes’ diets should be balanced in terms of proteins, fats, carbohydrates, and micronutrients (ACSM, 2016). However, there is an ongoing debate about the most optimal proportions of macronutrients in a diet leading to the reduction of excessive body fat. Undoubtedly, both high‐carb, low‐fat (HCLF) and high‐fat, low‐carb (HFLC) diets may change the measurements of body composition due to the reduction of body fat (Noakes & Windt, 2017). While these diets result in energy deficits, they may be applied to improve body composition of athletes. Properly balanced HFLC diets are considered to be safe and lead to successful control of body mass or reduction of the risk factors for cardiovascular diseases (Naude et. al., 2014; Johnston et al., 2014; Bueno et al., 2013; Ajala et al., 2013; Hu et al., 2012; Hession et al., 2009; Nordmann et al., 2006; Santos et al., 2012; Tobias et al., 2015; Sackner‐Bernstein et al., 2015). Moreover, HFLC diets seem to be popular due to the fast reduction of excessive body mass. However, it has to be noted that studies on the impact of high‐fat diets on body mass were short term, and their higher effectiveness regarding body mass loss results from the reduction of muscle glycogen and water loss, as opposed to from the reduction of body fat of test subjects (Yang & Van Italie, 1976). This results in the higher body mass reduction of the examined subjects in the short term. Nevertheless, in the long term, the changes of anthropometric measurements, as well as body composition, are comparable to the changes of the diets of higher carbohydrates intake, as was proved, among others, in the research using DEXA to measure body composition (Volek et al., 2009; Noakes, 2013; Mark et al., 2016). The reduction of body mass as a result of the implementation of negative energetic value diet does not only lead to the expected reduction of body fat but also adverse reduction of muscle mass. The importance of proteins in both diets is noteworthy, since, as far as a comparison of the effectiveness of the HFLC and HCLF diets in terms of the body mass reduction and body fat index is concerned, the examined participants often consume different amounts of proteins (the protein intake in HFLC diets is often higher) (Volek et al., 2004; Forsythe et al., 2008; Samaha et al., 2003). The increased number of proteins in a hypocaloric diet positively impacts the maintenance of fat‐free body mass, which might have influenced the obtained results of the research in terms of the comparison of the effectiveness of diets based on different fats and carbohydrates (Soenen et al., 2012). Hence, it is significant to keep a similar protein intake in diets based on different proportions of other macronutrients to assess their impact on body composition. Soenen et al. (2012) studied the importance of the impact of protein intake in a diet on body fat reduction. They proved that persons eating a high‐protein diet obtained a higher body mass reduction when compared to those following a low‐carbohydrate diet with a smaller number of proteins. It is worth emphasizing the current scarcity of research that would help address the question of which diet is more appropriate in terms of body mass reduction and the lowest impact on muscle mass reduction. The only methodologically well‐planned research work that is available at present shows that between groups following LFHC and LCHF diets for an appropriate time, there is no difference regarding their impact on body composition (Hall et al., 2016). Moreover, the impact of diets of different amount of fats and carbohydrates on body composition of athletes doing strength sports is unknown. Hence, this study aims to assess the impact of LFHC and LCHF diets on body composition of men doing strength sports with a healthy body mass, while maintaining the appropriate calorific value of a diet and protein intake.

## 2 METHODS

The research involved 55 men aged 19–35, with an average BMI of 24.01 ± 1.17 (min. 20.1, max. 26.1). The participants were required to be male, have been doing strength training for at least six months, at least three times a week, prior to the experiment, demonstrate the proper testosterone levels (300ng/dl‐800ng/dl), and the lack of chronic condition. The participants had been doing strength training for about 2 to 3 years. During the experiment, the participants were doing strength training adapted to their abilities and preferences for at least 3 days a week. Each training lasted about 70 min and was preceded by a 20‐min warm‐up. The participants mostly preferred to do a full‐body workout including basic exercises such as: barbell squat, deadlift, bench press, overhead press, chin‐up, hollow body, and push‐ups. The participants worked under the supervision of a personal trainer, and the training load was individually adapted to their abilities.

The study included three stages.

The first stage consisted in the recruitment of participants by employing a Cavi method, assessment of the daily eating pattern with the use of a 3‐day food intake record (Lewandowicz et al., 2015), anthropometric measures—height, body mass, arms, and waist and hips circumference, as well as the calculation of BMI (kg/m^2^). The dietary record was conducted on the basis of widely accepted and applied rules (FAO, 2018).

To provide the reliable estimates of food intake, participants were instructed about the principles of doing dietary record. The serving sizes were verified using the Polish “Atlas of food products and dishes portion sizes” (Szponar et al., 2000). The energy and nutritional value of diets were assessed using dietician software “Dieta 6.0” and the Polish base of the nutritional value of the products (Kunachowicz et al., 2005).

Moreover, the participants had their body composition examined with a bioelectrical impedance analysis (BIA) by employing Tanita MC‐780 P analyzer employing 4 electrodes. During the examination, the participants’ energy requirements were assessed on the level of BMR. The group was asked to avoid increased physical activity for 48 hr prior to the examination. Moreover, the participants did not smoke cigarettes and eat for 12 hr before the examination. The group was examined in the morning. BIA was measured by a qualified person, and the participants had to undergo it in their underwear. Also, the group was instructed to drink more than 2.5 liters of liquids for proper body hydration. According to EFSA recommendations (EFSA, 2010), before the BIA measurement, the participants were asked if they had noticed any syndromes of dehydrations. During the experiment, the participants were informed about the necessity to consume an additional amount of liquids so as to compensate the losses resulting from the increased physical activity.

The body height was measured with the use of a stadiometer. Waist, hips, arms, and thigh circumferences were measured according to standardized examination protocols published by National Health and Nutrition Examination Survey (NHANES, 2017). All of the measurements were taken by means of a professional meter (baseline tape measure, Tanita) with an accuracy of 0.1 cm.

To determine the level of physical activity (FAO, 2004), the recommendation of the Institute of Food and Nutrition, based on FAO/WHO [Human energy requirements], was applied. Finally, the participant's energy requirements were determined based on a basic metabolism rate in accordance with BIA measurement, which was then multiplied by a physical activity rate. The level of physical activity was assessed by using the physical activity questionnaire published by Johansson and Westerterp (2008).

In the second stage, the participants were asked to follow a basic diet for two weeks. The diet was designed according to the recommendations for healthy adults (Jarosz, 2012). The calorific value was in line with the energy requirements of each man participating in the research. Moreover, to provide the participants with the proper amount of energy, the calorific value of a diet was measured every 7 days while controlling the body mass. To assess research goal achievement, in the second and third stage, the participants made notes in their food diaries including the list of all the consumed products, meals, and drinks. In the event of a participant's failure to adhere to the daily schedule, he was immediately contacted and instructed to follow the prescribed diet. Moreover, the introduction of the basic diet aimed to determine the calorific value of an interventional diet, as well as the individual needs of the participants. Any changes in the body mass after two weeks were corrected by the application of the experimental diet. The percentage of carbohydrates in the diet was set at 55% of the calorific value, including added sugars to 10%. The percentage of energy from fats constituted 23%–35% of the calorific value of a diet, and the protein intake was set to 2 g per 1 kg of fat‐free body mass determined in BIA ^1^. The intake of vitamins and mineral components was determined on the basis of Polish food consumption standards (Jarosz, 2017). The participants received a 7‐day meal intake schedule that was individually customized to the preferences of taste. The proportions of ingredients were carefully modified, albeit in adherence to the goals of the diet schedule, to keep the participants motivated to complete their tasks. The participants received a detailed 7‐day nutritional plan with a detailed shopping list. After two weeks, we renewed the anthropometric measurements.

In the third stage, two interventional diets were applied for 12 weeks, shortly after the completion of the second stage. The caloric value of the interventional diets remained the same as was individually determined in the second stage and was aligned with the participant's energy requirements. The protein intake also remained unchanged and was set to 2g per 1 kg of fat‐free body mass. We used the principle of simple randomization to assign the participants to two groups: the low‐carb, high‐fat diet (LCHF) (*n* = 27) and low‐fat, high‐carb diet (LFHC) (*n* = 28). In the LCHF diet, carbohydrates constituted up to 40% (mean 38.7 ± 6.3%) of the caloric value of the diet, and fats complemented the caloric deficiencies (mean 40.2 ± 8.8%) of the caloric value of the diet. In the LFHC diet, fats constituted 21.7 ± 1.9% of the caloric value of the diet, and carbohydrates complemented the caloric deficiencies (mean 58.2 ± 4.8%) of the caloric value of the diet.

During the research, the participants were regularly examined by the head of the experiment and asked to submit a report including their body mass and anthropometric measurements (arms, waist, hips, and thigh circumference) every two weeks. Each participant received strict guidelines regarding filling the food diary. The notes included the list of all the consumed products, meals and drinks, using household measures (e.g., cups and spoons) and/or mass units (grams), and the duration of their consumption. The food diary also included information about the types of food supplementation and its daily amount. In the event of a participant's failure to adhere to the schedule, he was duly instructed to follow the guidelines. The proportions of ingredients were modified in adherence to the goals of the diet plan to keep the participants motivated to complete their tasks. At the beginning and end of the research, the measurements were conducted by the leader of the research. During the experiment, the anthropometric measurements were conducted by the participants, who had been trained to complete this task prior to the experiment. At the end of the third stage, the results of anthropometric measurements and the examinations of the body composition through BIA were collected. The composition of the general interventional diets is presented in Tables 1 and 2 presents detailed characteristics.

**TABLE 1:** General characteristic of interventional diets

Variable	LCHF group (*n* = 27) Mean ± *SD*	LFHC group (*n* = 28) Mean ± *SD*
Calorific value [kcal]	The calorific value was in line with the energy requirements of each man participating in the research	
Proteins [g]	2.0 g/kg fat‐free body mass (about 20% of energy)	
Fats in total [g]	40.2 ± 8.8%	21.7 ± 1.9%
Carbohydrates [g]	38.7 ± 6.3%	58.2 ± 4.8%

**TABLE 2:** Detailed characteristic of interventional diets

Variable	LCHF group (*n* = 27)	LFHC group (*n* = 28)
	Mean ± *SD*	Mean ± *SD*
Caloric value [kcal]	2.938 ± 153.9	2.687 ± 203.7
Caloric value [kcal/kg body mass]	35.36 ± 1.85	35.41 ± 2.68
Proteins [g]	142.5 ± 9.8	129 ± 16.2
Proteins [g/kg fat‐free body mass]	2.06 ± 0.13	2.01 ± 0.25
Fats in total [g]	131.3 ± 28.9	65 ± 5.8
Carbohydrates [g]	284.8 ± 46.9	391.2 32.9
Fiber [g]	41.7 ± 7	44.6 ± 6.9
Saturated fats [g]	33.4 ± 4.8	19 ± 2.9
Monosaturated fats [g]	67.9 ± 10.6	24.7 ± 3.5
Polysaturated fats [g]	25 ± 3.26	13.9 ± 2.6

**FIGURE 1:**
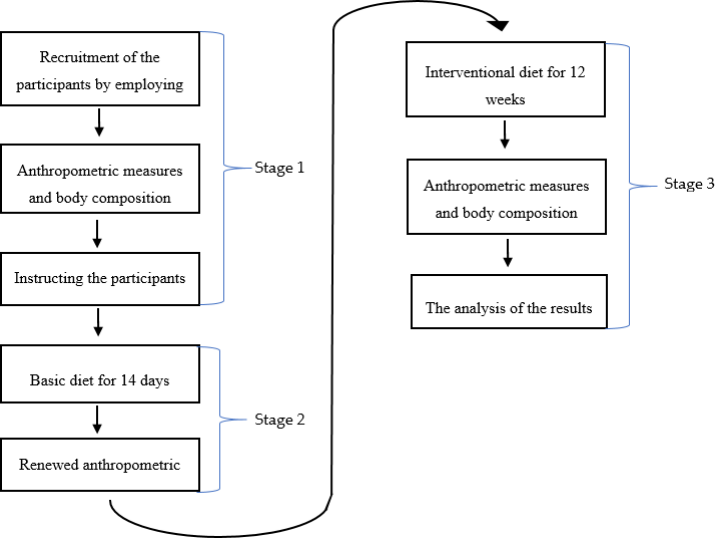
The scheme of the research

The local Ethics and Scientific Research on Humans Commission of Faculty of Human Nutrition and Consumer Sciences—SGGW (Warsaw University of Life Sciences) approved the research project (approval number: 17/2017). The scheme of the research is presented in Figure 1.

The quantitative study was conducted with the STATISTICA 13.3 Pl software (TIBCO Software Inc. 2017). Also, basic descriptive statistics were conducted on the basis of two groups. Due to the rejection of normal distribution hypothesis of most analyzed variables by Shapiro–Wilk *W* test, nonparametric tests were applied in this research: the Mann–Whitney *U* test (with continuity correction) and Wilcoxon matched‐pairs test, respectively. To reject the null hypothesis, *p* <.05, the liminal value was applied throughout the testing procedure.

## 3 RESULTS

Shortly before the start of the experimental diets, the median of participant's body mass (*p* = .00001) differed and amounted to 83.4 (60.4–95.4) kg in the LCHF group, and 76.6 (65–89) kg in the LFHC group. The median of BMI in the LCHF group amounted to 24.7 (20.4–26.2) kg/m^2^ and 23.9 (20.1–25.6) kg/m^2^ in the LFHC group. The differences in BMI between groups were statistically significant (*p* = .03). WHR in both groups was within standards, and its median was 0.95 (0.9–1.0) for the LCHF group, and 0.97 (0.84–1.03) for the LFHC group. The median of arm circumference in the LCHF group for the right and left arm was 36 (29–39.5) cm and 35.5 (29–39) cm, respectively. In the LFHC group, the circumferences were significantly smaller (*p* = .001), and their median was 33 (29–38) cm for both arms. In the LCHF group, the median of thigh circumference for the right and left thigh was 56.5 (51–61) cm and 57 (51–61) cm, respectively. In the LFHC group, the circumferences did not significantly differ (*p* = .2) and amounted to 56 (51–61) cm and 55.5 (50–61) cm, respectively. The median of the absolute amount of body fat mass significantly differed between groups (*p* = .01) and in the LCHF group was 14 (6.7–19.8) kg, while in the LFHC group was 12.2 (5.1–16.3) kg. On the other hand, the participants’ body fat levels did not differ among groups (*p* = .5) and amounted to 17,1 (9,8–21,2) % in the LCHF group, and 16,2 (7,5–22,6) % in LFHC group. Also, the participant's body hydration levels did not significantly differ among groups (*p* = .07), and in the LCHF group, the median was 59.2 (55–64.7) %, whereas in the LFHC group it was 60.9 (55.9–69.9) %. Other features of the groups are presented in Table 3.

**TABLE 3:** Anthropometric measures and body composition at the early stage of the research

Variable	LCHF group (*n* = 27)		LFHC group (*n* = 28)		*p* *
	Mean ± *SD*	Median (min‐max)	Mean ± *SD*	Median	
(min‐max)					
Age [years]	28.33 ± 3.01	29 (23–34)	26.71 ± 3.56	26.5 (19–35)	.06
Height [cm]	184.16 ± 5.66	185 (171–195)	178.71 ± 5.07	178.3 (168–192)	.002
Body mass [kg]	83.07 ± 7.4	83.4 (60.4–95.4)	75.88 ± 4.79	76.6 (65–89)	.00001
BMI [kg/m^2^]	24.34 ± 1.07	24.7 (20.4–26.1)	23.7 ± 1.2	23.9 (20.1–25.6)	.03
Waist circumference [cm]	85.2 ± 4.2	86 (75–93)	81.14 ± 5.63	8.5 (70–90)	.01
Hip circumference [cm]	89.13 ± 4.66	89 (76–96)	84.26 ± 5.87	85 (72–95)	.002
WHR	0.95 ± 0.02	0.95 (0.9–1.0)	0.96 ± 0.04	0.97 (0.84–1.03)	.07
Right arm circumference [cm]	35.25 ± 2.08	36 (29–39.5)	33.48 ± 2.1	33 (29–38)	.001
Left arm circumference [cm]	35.20 ± 2.01	35.5 (29–39)	33.44 ± 2.20	33 (29–38)	.001
Right thigh circumference [cm]	56.35 ± 2.28	56.5 (51–61)	55.69 ± 2.58	56 (51–61)	.2
Left thigh circumference [cm]	56.2 ± 2.24	57 (51–61)	55.42 ± 2.83	55.5 (50–61)	.2
Body fat mass [kg]	13.9 ± 3.21	14 (6.7–19.8)	11.76 ± 3.06	12.2 (5.1–16.3)	.01
Fat content [%]	16.33 ± 3.24	17.1 (9.8–21.2)	16.98 ± 9.66	16.2 (7.5–62.6)	.5
Free fat mass [kg]	69.17 ± 5.76	70.4 (53.7–78.9)	64.12 ± 3.87	64.2 (56.4–73.8)	.002
Body hydration [%]	59.46 ± 2.78	59.2 (55–64.7)	61.18 ± 3.30	60.9 (55.9–69.9)	.07

* Mann–Whitney *U* test.

It is worth emphasizing that after 12 weeks of intervention, the changes between the groups in body weight, body composition, and its circumference did not differ statistically significantly. However, significant changes in body composition within the studied groups were observed. Following the 12‐week‐long experiment based on the LCHF diet, the participants’ body mass significantly decreased by 1.5% on average (*p* = .01), while BMI decreased by about 1.3% (from 24.7 (20.4–26.1) kg/m^2^ to 24.3 (20.3–5.8) kg/m^2^); the change was close to the limit of statistical significance (*p* = .06)). In the LFHC group, the parameters did not significantly change, except for the hip circumference measurement (Table 3). In the LCHF group, arm circumference significantly decreased by 1.4%, whereas in the LFHC group, it increased by 0.6–0.8%. In both groups, no significant change in thigh circumference was noted. In the LCHF group, the amount of body fat (from 14 (6.7–19.8) kg to 12.7 (3.9–19.2)) decreased by 8.6% (*p* = .01) (in the absolute value of 1.2 kg), which corresponded to its reduction in body composition by 1.02 percentage point. In the LFHC group, in turn, body fat mass also significantly decreased by 1.5% (*p* = .01) (of 0.4 kg), but the change was smaller than in the LCHF group, and no significant changes in relative body fat in participant's body were found. In both groups, no significant changes in free fat mass were demonstrated. However, it has to be mentioned that in the LCHF group, a slight increase (by 0.1%) in participants’ body hydration was noted. Other contingencies and changes in this group are presented in Table 4.

**TABLE 4:** Anthropometric measures after 12 weeks of following the interventional diet and the proportion of changes

Variable	LCHF group					LFHC group					Istotność zmian między grupami P
	Mean ± *SD*	Median (min‐max)	Change [%]	Change*	*p* **	Mean ± *SD*	Median (min‐max)	Change [%]	Change*	*p* **	
Body mass [kg]	81.83 ± 7.36	81.3 (60.2–94.1)	‐ 1.5%	−1.24	.01	76.07 ± 4.82	76.8 (66–90)	0.3%	0.19	.5	0.064***
BMI [kg/m^2^]	24.03 ± 1.23	24.3 (20.3–25.8)	‐ 1.3%	−0.31	.06	23.81 ± 1.14	23.8 (21–25.8)	0.5%	0.11	.3	0.10***
Waist circumference [cm]	83.09 ± 5.45	86 (75–93)	‐ 2.5%	−2.11	.01	80.21 ± 4.99	80.5 (71–90)	‐ 1%	−0.93	.06	0.66***
Hip circumference [cm]	87.92 ± 4.98	89 (76–96)	‐ 1.3%	−1.2	0.07	83.71 ± 5.9	85 (72–95)	‐ 0.7%	−0.55	.01	0.95***
WHR	0.93 ± 0.38	0.95 (0.9–1.0)	‐ 1.4%	−0.01	.04	0.95 ± 0.04	0.95 (0.85–1.03)	‐ 0.3%	−0.003	.5	0.73***
Right arm circumference [cm]	34.75 ± 2.16	35 (29–40)	‐ 1.4%	‐ 0.5	.01	33.66 ± 2.22	33 (30–39)	0.6%	0.23	.4	0.054****
Left arm circumference [cm]	34.72 ± 2.22	35 (29–40)	‐ 1.4%	‐ 0.28	.02	33.71 ± 2.3	33 (30–39)	0.8%	0.27	.1	0.052****
Right thigh circumference [cm]	56.46 ± 2.78	57 (50–62)	0.2%	0.11	.6	55.64 ± 2.64	56 (51–62)	‐ 0.1%	0.05	1	0.57****
Left thigh circumference [cm]	56.57 ± 2.82	57 (50–62)	0.7%	0.37	.09	55.48 ± 2.89	56 (51–62)	0.2%	0.1	0.8	0.79***
Body fat mass [kg]	12.69 ± 3.54	12.7 (3.9–19.2)	‐ 8.6%	−1.2	.01	11.37 ± 2.8	12.1 (5.6–16.8)	‐ 1.5%	−0.4	.01	0.28***
Fat content [%]	15.31 ± 3.59	17.1 (9.8–21.2)	‐ 6.1%	−1.02	.01	16.59 ± 9.87	15.65 (8–64.3)	‐ 0.9%	‐ 0.39	0.3	0.27***
Free fat mass [kg]	69.14 ± 5.51	69.6 (56.3–79.4)	−0.2%	−0.03	.91	64.7 ± 3.69	64 (57.3–71.7)	1.1%	0.64	.16	0.15***
Body hydration [%]	59.5 ± 9.19	60.1 (55.1–71.8)	0.1%	0.04	.01	61.68 ± 2.62	61.05 (56.4–68.5)	0.9%	0.5	.1	0.34***

* Mean change in absolute values.

** Wilcoxon matched‐pairs test.

*** Mann–Whitney *U* test.

**** Student's *T* test.

## 4 DISCUSSION

Male participants of the examination were randomly attached to two interventional groups. The age of men in both groups did not significantly differ. Men in the LCHF group demonstrated higher BMI, absolute fat, and free fat mass than the men in the LFHC group. However, their body composition did not significantly differ, and the relative amount of body fat was similar. Following the 12‐week‐long experiment based on interventional diets, the participants eating a high‐fat diet demonstrated a significant reduction of body mass in absolute values, which led to a small change in their BMI, which, however, did not reach statistical significance.

The caloric value of the diet was adjusted to the individual needs of each participant. Due to the higher body weight, the participants in the LCHF group consumed more energy in their diet. However, if we look at the relative values expressed in the amount of energy intake per kilogram of body weight, the values between the groups do not differ statistically, and amount to 35.36 ± 1.85 kcal per kg of body weight in the LCHF group, and 35.41 ± 2.68 kcal per kg b.w. in the LFHC group.

It is worth noting that in this study, despite the observed significant changes in the body composition within the groups, no differences in the anthropometric parameters between the groups were found, when comparing the high and low intake of carbohydrates in the diet over the course of 12 weeks, and when the energy requirements of the diet were adjusted to the needs of participants. Our results indicate that the energy intake of the diet and the level of physical activity are the main factors affecting body composition in people doing strength training.

In the case of the group eating a low‐fat diet, no change of body mass, BMI, was found. The reduction of body mass in the LCHF group resulted from the reduction of body fat. Also, in this group, free fat mass did not change. This resulted in a relative reduction of the participant's body fat levels. In the LFHC group, a notable reduction of body fat content was found; however, it did not impact the levels of free fat mass. The calorific value in both diets covered the individual needs of the men. However, it has to be noted that in both groups, no impact of the diets on free fat mass was found. Unfortunately, there are no studies concerning a similar group following diets of different fats and carbohydrates content. The impact of diets of high‐fat content on overweight men was studied by Hall et al. (2016). They noticed the reduction of body mass of participants after four weeks of following an isocaloric ketogenic diet, which was particularly notable in the first days. The authors stated that the observed body mass reduction was mainly related to the reduction of glycogen in muscles and water content in the participants’ bodies. The reduction of water in a body is also confirmed in another research in which low‐carbohydrates diets were applied. Their authors pointed to the fact that the reduction of participants’ body mass was not the result of only this factor (Volek et al., 2009; Mark et al., 2016; Hussain et al., 2012; Dashti et al., 2004; Noakes, 2013).

However, contrary to the conclusions made in the abovementioned studies, in our research, after the 12‐week‐long experiment based on the LCHF diet, we noted a slight increase in participants’ body hydration. Nevertheless, it is noteworthy that energy from fat in other studies was higher, and energy from carbohydrates was lower than in the LCHF diet, which was applied in this research. In our experiment, the applied high‐fat diet provided about 40% of the calorific value from fats, which is an acceptable amount in the proper nutrition recommendations. Moreover, it has to be noted that the participants found it challenging to follow a diet low in carbohydrates, which led to deviations from the diet and increase in the size of meals (Close et al., 2016). Also, excessive reduction of carbohydrates intake by athletes doing strength sports, or high‐intensity interval training, may negatively impact exercise capacity (Close et al., 2016; Forbes et al., 2020).

The research on the impact of diets of different proportion of fat content to carbohydrates on body composition shows their similar effectiveness (Foster et al., 2010; Gardner et al., 2007; Shai et. al., 2008). However, it has to be noted that in this research, diets of different levels of macronutrients and similar calorific value were applied. These diets were of lower energetic value than the participants needed. The aim of applying these diets was body mass reduction. Undoubtedly, energy deficits are the essential indicator of reduction of excessive body mass. Although the low‐carbohydrate diet may effectively lead to the fast reduction of body mass, in the long term, it does not have a more significant effect than the diet of a higher amount of carbohydrates. On the other hand, these findings show that it is possible to modulate diet content while including the nutritional preferences of the athletes.

Furthermore, reports indicate a possible occurrence of adverse effects of following high‐fat diets, especially when the reduction of carbohydrates is very high. Among the possible conditions, one may find headaches, tiredness, and muscle cramps (Westman et al., 2007). However, it has to be noted that the symptoms were temporary and occurred only within the period of adaptation to increased fat intake. Nevertheless, these symptoms necessitated the monitoring of the participants’ mood. In our research, we did not note any adverse effects regarding mood and an increase in muscle aches of the applied diets. This suggests that a minor alteration of carbohydrates and fats intake does not lead to adverse effects.

## 5 CONCLUSION

To sum up, one can say that the observed changes in the body composition, although significant within the groups, did not differ significantly when compared between groups. Our results show that in strength‐training men, who have their energy and protein requirements covered, different amounts of carbohydrate and fat intake do not affect the changes in body composition. Thus, the observed changes in anthropometric parameters could result mainly from the training process. Nevertheless, further research is needed to assess the impact of diets with high and low‐fat intake on the anthropometric parameters of training men.

Both diets allowed maintenance of the free fat mass within 12 weeks of the experiment. However, in practice, first of all, eating habits and individual preferences of the athletes should determine the choice of eating routine aiming at the change of anthropometric parameters indicators.

## CONFLICT OF INTEREST

The authors declare that they do not have any conflict of interest.

## ETHICAL STATEMENT

The local Ethics and Scientific Research on Humans Commission of Faculty of Human Nutrition and Consumer Sciences—SGGW (Warsaw University of Life Sciences) approved the research project (approval number: 17/2017).
